# Editorial: Today's Nutrition and Tomorrow's Public Health: Challenges and Opportunities

**DOI:** 10.3389/fphar.2016.00034

**Published:** 2016-02-24

**Authors:** Irene Lenoir-Wijnkoop, Iñaki Gutiérrez-Ibarluzea, Dominique J. Dubois

**Affiliations:** ^1^Department of Pharmaceutical Sciences, Utrecht UniversityUtrecht, Netherlands; ^2^OSTEBA - Basque Office for Health TechnologyVitoria-Gasteiz, Spain; ^3^PHARMED, Université Libre de BruxellesBrussels, Belgium

**Keywords:** nutritional physiological phenomena, ecosystems health, contextual research, health technology assessment, sustainable health infrastructures

Scientific research in the field of nutrition and health has made great strides in recent years. Food-related investigations cover a broad range of topics in close relation with many other health-oriented disciplines. This E-book elaborates on the relevance of putting diet and food habits at the heart of strategies aiming to improve the global health status of the general public, and on the need of appropriate methodological approaches to conduct meaningful and reliable public health- and economic outcome evaluations.

An emerging issue in the field of nutrition is the important role of our food on physiological homeostasis and resilience of the human body, as highlighted by Tremblay et al. The authors shed a new light on the obesity issue by addressing various functionalities of the adipose tissue, looking beyond its traditional function of excess energy storage. The multifactorial interplay between biological, environmental, and socioeconomic determinants may provide a plausible explanation of the obesity epidemics. The documented examples include:

- short sleep duration as a predictive variable for weigh gain
- the shift from physical work to cognitive efforts leading to a decreased energy expenditure but an increased energy intake, possibly due to an impaired satiation process
- uptake of chemical pollutants by the adipose tissue, which allows protection of other body tissues, but seems associated with a higher risk to develop a metabolic syndrome.

Further, research on the involvement of the adipose tissue in many regulatory processes will allow to better understand how nutrition-related body homeostasis should be taken into account in relation to the global constraints of sustainable development.

Another environmental key player is the microbiome ecosystem, an integrated part of the human organism. Ganesh and Versalovic present an insightful review on the role of our gut microbiota, and its composition, on health and disease. On the basis of a careful review of the literature with a focus on immune regulation, the authors address the direct and indirect interplay between the gastro-intestinal tract, its commensals and nutrients, both ingested and produced through metabolic processes. Bacterial-derived metabolites are known to influence host immune responses, while dysregulation of the related cellular and molecular pathways may affect the gut functionalities and increase the host's susceptibility to immune-mediated pathologies. This underlines the importance of beneficial micro-organisms in regulating the homeostasis of the human body. Further, investigations of these mediating properties can have important implications for the development of cost-effective preventative interventions to manage the increasing number of gastro-intestinal and metabolic disorders.

When studying the effects of food on human beings in their macro- or micro-environment, the citizen him- or herself—whether considered as being healthy, part of an at risk population or a patient—is obviously an “interfering” factor who impacts the larger public health context. Based on these considerations, Segal and Opie make a strong plea for implementing comprehensive nutrition strategies to reduce the diet-related disease burden. Such strategies need to incorporate both public health approaches and expanded publicly funded dietetic services. Multi-component strategies are proposed which include social marketing, regulatory restrictions on advertising of junk food/drinks, punitive taxes on unhealthy foods, suitable food labeling and publicly funded dietician services. Dietetic services are suggested to be part of core health service delivery and funded at a level that supports access to individualized dietetic services. Adopting such strategies may lead to substantial improvements in diet quality, better health, and wellbeing and lower healthcare costs.

A similar line of thought can be found in the work by Lenoir-Wijnkoop et al. who propose a health economic framework to assess short-term costs of maternal overweight, gestational diabetes, and related macrosomia. The authors point out that, in spite of the indubitable impact of overweight and obesity on public health, not much focus has been put on their social and economic consequences. The calculation of the costs associated to maternal overweight, gestational diabetes, and related macrosomia indicated that health expenditures are considerable. In fact, the conservative estimation of the decision analytical techniques based model, using US costing data, provided an annual cost close to 2 billion US$. These results underpin the hypothesis that public health interventions devoted to lifestyle, diet, and physical activity not only improve health status, but also have measurable social and economic consequences. The long-term potential of these interventions should be considered in the prevention of non-communicable diseases and healthcare systems sustainability.

An important element that might help to improve the efficacy of the above mentioned nutrition strategies and interventions, is introduced by Ruano-Rodríguez et al. They discuss the increasing need for “customer” self-reported outcome measures of Health-Related Quality of Life (HRQoL) in the field of nutrition, including unhealthy lifestyles and dietary habits. A literature research revealed that the generic SF-36® is the most frequently used health status questionnaire. However, very few validated HRQoL measurement instruments are available to address the specific context of preventative dietary interventions. Contextual diet-specific HRQoL measures are needed for evaluating the impact of diet habits on daily life functioning and wellbeing.

Context is indeed key for obtaining meaningful research outcomes on nutrition and health. Therefore, future progress in this area will also depend on our ability to integrate the nutritional dimension in a societal perspective as brought up by Poley. He addresses the challenges, opportunities of health technology assessment (HTA) in the field of nutrition interventions and its role in policy making. Nutrition and HTA used to be two worlds apart. However, consensus is growing that HTA can provide useful tools to substantiate the positive impact of nutrition on public health, despite the differences with HTA of disease treatment. For example, food products are typically paid “out-of-pocket” by the consumer rather than by a third-party payer. Contextual research should be able to address the methodological issues. In addition, better understanding of the policymakers' needs and efficient integration of HTA results into policy making will also be needed.

This idea is supported by Gutiérrez-Ibarluzea and Arana-Arri who revise the status of nutrition and its assessment by HTA specialists. Although HTA has an extensive scope and a wide range of technologies that could be considered under its remit, HTA has classically focused on the evaluation of medicinal products, medical devices, and their consequences for the healthcare systems. This overview shows that HTA specialists, mainly from high income countries, are increasingly interested in evaluating nutrition and its consequences. Well-established institutions have even suggested methodological approaches to the assessment of nutrition and have reported the utility of systematic reviews and economic evaluations to address the impact of nutrition. That notwithstanding, the evaluation of nutrition, as is the case of other public health interventions, requires the refinement of some commonly used measures and standards, such as the Quality Adjusted Life Years (QALY) paradigm for economic evaluations.

Two other studies illustrate how traditional and widely consumed foodstuffs can have a considerable influence on daily health and nutrition-related public health issues. Abdullah et al. performed an economic evaluation on the potential cost-savings to the Canadian healthcare system of two diet related diseases, type 2 diabetes (T2D) and cardiovascular disease (CVD), by increasing the intake of dietary fiber by adults. The authors performed a three-step cost-of-illness analysis to identify the percentage of individuals expected to increase their fiber intake, the potential reduction rates of T2D and CVD and the related annual savings due to this reduction. The study findings show that 1 g increase per day in universal fiber consumption would result in annual cost-savings of up to 51 million CAD$ for T2D and 92 million CAD$ for CVD. Even in the most pessimistic scenario the cost-savings remained substantial. This study shows that strategies aiming to improve dietary fiber intake as part of a healthy diet could have a significant direct impact on healthcare and related costs.

And last but not least, Glanville et al. describe the outcomes of a scoping review of the volume of available evidence on the health effects of conventional yogurt consumption. Studies considered eligible for the scoping review were epidemiological studies, cohort studies, open label studies, and randomized controlled trials (RCTs). Two hundred and thirteen studies were identified as relevant to the scoping question. The review identified a number of outcomes for which there exists substantial primary evidence that may be suitable for systematic review and potentially meta-analysis.

The 103 pages of “Supplementary Materials” contain a wealth of information on the “Full Search Strategies” and summary tables on study outcomes in bone health, weight management, metabolic health, cardiovascular health, gastrointestinal health, cancer, diabetes, and other diseases. The Supplementary Tables for this article can be found online at: http://journal.frontiersin.org/article/10.3389/fphar.2015.00246.

In summary, this Research Topic of Frontiers underlines the strong intertwinement of nutrition research with other scientific, social, economic, and political research fields. The real-world setting further adds to the complexity due to the central place of the citizen who appears to be an active, although often unconscious, influencer of his surroundings. Concerted efforts by all parties involved will be mandatory to obtain stronger evidence-informed insights to guide decisions on convincing strategies for efficient and sustainable changes in dietary habits and nutrition-related lifestyle. There is a lot at stake!

## Author contributions

The two co-authors of this editorial, DD and IG, provided each part of the short summaries that refer to the full paper contributions received and published in this RT. When I had written the overall Editorial DD reread the proposed text which we then validated together before I finalized the document.

### Conflict of interest statement

The authors declare that the research was conducted in the absence of any commercial or financial relationships that could be construed as a potential conflict of interest.

The author DD and handling Editor declared their shared affiliation, and the handling Editor states that the process nevertheless met the standards of a fair and objective review.

